# Longitudinal and Combined Smartwatch and Ecological Momentary Assessment in Racially Diverse Older Adults: Feasibility, Adherence, and Acceptability Study

**DOI:** 10.2196/69952

**Published:** 2025-04-08

**Authors:** Sophia Holmqvist, Marina Kaplan, Riya Chaturvedi, Haochang Shou, Tania Giovannetti

**Affiliations:** 1 Department of Psychology and Neuroscience Temple University Philadelphia, PA United States; 2 Department of Biostatistics, Epidemiology, and Informatics University of Pennsylvania School of Medicine Philadelphia, PA United States

**Keywords:** cognitive impairment, smartwatch, longitudinal monitoring, ecological momentary assessment, aging

## Abstract

**Background:**

Due to the rising prevalence of Alzheimer disease and related dementias, easily deployable tools to quantify risk are needed. Smartphones and smartwatches enable unobtrusive and continuous monitoring, but there is limited information regarding the feasibility, adherence, and acceptability of digital data collection among racially diverse older adults.

**Objective:**

This paper examined the feasibility, adherence, and acceptability of a 4-week combined smartwatch monitoring and ecological momentary assessment (EMA) study in a racially diverse sample of older adults.

**Methods:**

A total of 44 older adults (aged ≥55 y) with either mild cognitive impairment or healthy cognition completed an informed consent comprehension quiz, baseline cognitive testing, training regarding digital data collection, and questionnaires. Participants were instructed to wear a Garmin Vivosmart 4 smartwatch for 23 h/d for 4 weeks, sync 2 smartphone apps (Garmin and Labfront) daily, and complete a daily EMA survey with automated prompts for surveys and charging. Training time, smartwatch adherence (eg, wear time), daily EMA survey response rate, and performance on the consent quiz were quantified. Associations between feasibility and adherence metrics and participant factors were evaluated. Self-reported usability of the apps and smartwatch was collected at study end.

**Results:**

Consent comprehension quiz scores were high (mean 97.33%, SD 6.86% correct), and training sessions lasted on average 17.93 (SD 6.89) minutes. During the 4-week study, participants wore the smartwatch for an average of 21 h/d (SD 1.53) and showed an average response rate of 94% (SD 9.58%) to daily EMA surveys. In unadjusted bivariate analyses, age, race, and cognition were associated with feasibility and adherence measures, but only age and race remained significant in multivariate models. After accounting for all participant factors, older age was a significant predictor of longer training time, and Black race was a significant predictor of lower daily wear time. On the usability survey, all participants (45/45, 100%) indicated willingness to participate in future smartwatch studies, >80% (37/45) had a positive experience, and >90% (41/45) were satisfied with smartphone app syncing.

**Conclusions:**

Smartwatch monitoring, requiring daily wear, smartphone syncing, and daily EMA survey completion, is highly feasible in older adults because adherence to daily wear and EMA surveys was high, as was general satisfaction on usability surveys. Although older participants may require more training on smartwatch and smartphone procedures and automated prompting during the study period, longitudinal monitoring with the Garmin Vivosmart 4 smartwatch and Labfront app is acceptable and feasible for collecting nearly continuous data in Black and White older adults, including those with mild cognitive impairment and those without.

## Introduction

### Background

With the prevalence of Alzheimer disease (AD) and AD and related dementias (ADRD) increasing alongside the aging population and the availability of new treatments, there is a need to quantify risk and detect cognitive decline at the earliest stage [1]. It is estimated that only a small minority of older adults with mild cognitive decline are accurately identified early [2], missing an opportunity for early intervention. Missed or delayed diagnoses are even more common in Black and African American older adults, who are also at increased risk for AD/ADRD [3]. Personal digital devices such as smartwatches have been proposed as a potential tool for the early detection of AD/ADRD. Although recent data show that smartwatches have been increasingly adopted by older adults [4], the utility of personal digital tools for AD/ADRD monitoring is dependent upon consistent smartwatch use and syncing with associated software. This study examined the feasibility of and adherence to daily smartwatch wear and ecological momentary assessment (EMA) survey completion in a racially diverse sample of older adults enrolled in a 4-week study. We also investigated older adults’ comprehension of study details that are relevant to informed consent. The participant characteristics associated with smartwatch adherence and the comprehension of consent were explored to optimize adherence and ethical research conduct in future studies.

Observable yet subtle cognitive, sensory, and motor changes precede and predict the clinical manifestations of AD/ADRD [1,5] and may be measured by sensors in commercially available smartwatches; for example, the Garmin smartwatch has been validated against conventional clinical measures of sleep [6], activity [7,8], and heart rate variability [7], which are associated with dementia risk [9-11]. The Garmin Vivosmart 4 smartwatch also has shown positive results in a 2-week feasibility study of stress in adults undergoing psychotherapy [12]. Thus, digital measures of stress, low heart rate variability, reduced physical activity, and sleep alterations have great potential as low-cost digital biomarkers for AD/ADRD that may be measured continuously, longitudinally, and passively, requiring relatively little effort.

EMA questionnaires delivered directly to participants via their personal digital devices enable high-frequency data collection in the participants’ natural setting. EMA can be useful for contextualizing passive metrics [13]; for example, EMA data may facilitate the interpretation of atypical data as clinically significant or not (eg, elevated heart rate due to a cardiac event vs a day at an amusement park). EMA data also provide insight into participants’ immediate perceptions and mood states without the confounds of recall bias [14]. There is potential for passive and EMA data collection via personal digital devices, but limited data exist on the feasibility and adherence of device use for health monitoring in older adults. In addition, the extent to which cognitive ability level influences adherence is not known. Adherence to daily EMA surveys and key tasks, such as wearing, charging, and syncing digital devices, is crucial for valid and reliable measurement and requires cognitive resources. This raises an important question: are those who could benefit the most from digital health monitoring more likely to show poor adherence?

Our review of the literature identified few studies on the feasibility and adherence of smartwatch use in digital health research. The results of the available studies suggest several important moderating factors, including the study procedures and participant factors. In-person studies and study designs that include interaction with a member of the study team show much higher retention and adherence than fully remote studies [15]. Adherence also varies depending on task demands, with different adherence rates for smartwatch wear versus syncing versus EMA completion. Adherence to wearable device use, which is relatively less demanding, versus the completion of daily EMA surveys has been estimated to be approximately >90% and 70%, respectively, in studies that are not fully remote [16-19].

Participant factors also play a role in retention and adherence. Although age is commonly considered a barrier to the adoption and acceptability of smartwatches due to low digital literacy [20], empirical data aggregated across several studies of smartwatch data show that older age was associated with greater retention [15]. A study requiring older adults with healthy cognition to wear a Fitbit device for 30 days during waking hours reported high rates of adherence to daily wear (89% of study days) and syncing (85% of study days) [21]. Other studies have demonstrated the effects of race, sex, and memory ability on adherence such that White participants [15], women [22], and people with better memory abilities [21] are more adherent when asked to engage in digital health research studies that involve interacting with a smartphone app.

In addition to the importance of learning about smartwatch adherence and feasibility, it is also crucial to understand older adults’ comprehension of informed consent procedures for studies using novel technology. With the large amount of health data collected from a smartwatch and the need for long-term data storage, it is vital for older adult participants to fully understand data privacy and security limitations and the associated risks of study participation. Older adults also may be more wary of sharing personal health information in the context of digital health research. To assess older participants’ understanding of informed consent procedures, interactive quizzes have been developed for use in fully remote studies [23]. For this study, which included face-to-face interactions with members of the study team, a 10-item comprehension quiz was modeled after a quiz used by Hackett et al [13] that was designed according to published guidelines [24,25]. The consent quiz focused on the novel technology used in the study (eg, where participants’ data are stored and the right to request digital data to be deleted at any time). Hackett et al [13] reported high accuracy rates on a similar quiz in a sample of older adults and significant associations between quiz scores and education level. Quiz scores also varied by race, with Black participants scoring lower than White participants.

### Objectives

In this study, feasibility and adherence were evaluated over a 4-week monitoring period that required wearing the smartwatch daily (23 h/d), charging it, syncing apps, and completing EMA surveys. Feasibility and adherence were assessed by tracking participant retention and measuring the following: (1) time required to complete study training on syncing apps and completing EMA surveys, (2) the comprehension of informed consent information, (3) daily smartwatch wear time, (4) adherence to smartwatch daily wear, and (5) the completion of daily EMA surveys. We also investigated associations between feasibility and adherence measures and participant characteristics (demographics, cognition, and self-reported functional and cognitive decline). Finally, we explored participants’ perceptions regarding the usability of the Garmin Vivosmart 4 smartwatch and the study-related apps (syncing process). To address these aims, we recruited a racially diverse sample of older adults, including those with mild cognitive impairment (MCI) and those without. It was hypothesized that older age, worse cognitive function, and greater self-reported functional decline would be associated with lower daily wear time, adherence to wear time, and survey completion, as well as lower comprehension of consent.

## Methods

### Participants

A total of 47 community-dwelling older adults aged ≥55 years classified as having healthy cognition or MCI were recruited from the Temple University Cognitive Neuropsychology Laboratory cohort in Philadelphia, Pennsylvania. This cohort includes >200 adults who have participated in ongoing federally funded studies since 2020 and represent the racial, ethnic, and economic diversity of the Philadelphia area. Participants met the following inclusion criteria: (1) oral and written fluency in English (to complete study questionnaires and measures) and (2) no history of large vessel stroke, Parkinson disease, major traumatic brain injury, seizures, schizophrenia, or significant neurological conditions other than dementia. The exclusion criteria included (1) current psychiatric disorder (eg, bipolar disorder or major depressive disorder), (2) intellectual disability, and (3) severe motor and sensory deficits precluding the use of a computer touchscreen. Participants from the laboratory cohort were contacted and screened for the following inclusion criteria for this study: (1) aged ≥55 years, (2) cognitive status classified as healthy or MCI, (3) current smartphone user (Android or IOS), and (4) not currently wearing a smartwatch or willing to wear only the study smartwatch during the study period. The exclusion criteria included (1) a diagnosis of dementia and (2) scheduled surgery or travel during the 4-week study period. Approximately 80 participants from the laboratory cohort were contacted as part of the recruitment efforts. The most common reasons for nonparticipation were not having a smartphone or a lack of interest in completing a month-long study.

Of the 47 participants recruited for this study, 1 (2%) was excluded because they required a walker for mobility, which precluded accurate step count recording, and 1 (2%) was excluded from the analyses of feasibility and adherence because their smartphone malfunctioned, and they required the monitoring period to be extended. Moreover, 1 (2%) of the 47 participants dropped out after 1 day, citing discomfort from the smartwatch band being too tight. Thus, of the 47 enrolled participants, 3 (6%) were excluded, resulting in a final sample of 44 (94%) participants for the feasibility and adherence analyses. The participant who extended their monitoring period because of the smartphone malfunction was retained for the usability analyses.

### Ethical Considerations

This study was approved by the Temple University Institutional Review Board (Protocol Number: 29712). All participants provided written informed consent and received a US $50 gift card as compensation for their time. Data storage and procedures were Health Insurance Portability and Accountability Act–compliant and only approved study personnel had access to study data.

### Procedures

#### Overview

Participation in the study involved 3 phases. First, participants completed an initial in-person study visit lasting 2 to 4 hours, which included comprehensive cognitive testing, training for the study, and questionnaire completion. Second, participants wore the smartwatch for 4 weeks, answered daily questions on their smartphone, and synced the smartwatch with a smartphone app once a day. Finally, all participants completed a debriefing session. More details of each study phase are provided in the following subsections. Textbox 1 shows the study timeline. The cognitive tests administered during study visit 1 are listed in Table 1.

**Study timeline.**

**Study visit 1**

- Informed consent and comprehension quiz
- Installation of Garmin and Labfront apps
- Study training on syncing and daily survey
- Questionnaires and cognitive testing

**4-wk monitoring period**

- Participants engage in daily activities as usual
- Wear smartwatch for 23 h/d
- 9-question ecological momentary assessment survey once a day
- Sync Labfront and Garmin apps once a day to securely collect and transmit deidentified data

**Study visit 2**

- Uninstall apps and unpair smartwatch
- Debriefing questionnaire
- Payment of US $50

**Table 1:** Cognitive domains and corresponding neuropsychological measures.

Cognitive domains	Neuropsychological measures
Attention	Trail Making Test part A [26] Digit Span Forward [27]
Executive function	Trail Making Test part B [26] Digit Span Backward [27]
Language	Letter fluency (S and P) and category fluency (animals) [28] Boston Naming Test, 30-item version [29]
Processing speed	Salthouse Letter Comparison Test [30] Salthouse Pattern Comparison Test [30]
Episodic memory	Hopkins Verbal Learning Test: immediate recall, delayed recall, and recognition trial [31] Brief Visual Memory Test–Revised: immediate recall, delayed recall, and recognition trial [32]

#### Initial Study Visit

Participants completed the following during study visit 1, which lasted 2 to 4 hours: (1) informed consent and 10-item consent comprehension quiz on the specific features of the study (eg, data security and privacy; refer to Multimedia Appendix 1 for the quiz items); (2) gold standard cognitive tests (Table 1) and questionnaires; (3) installation and configuration of study apps and training on wearing a Garmin Vivosmart 4 smartwatch daily (23 h/d of wear; 1 h/d of charging time) for 4 weeks, syncing the smartwatch, and completion of a daily EMA survey. At the end of the first study session, a follow-up visit was scheduled for a date at least 4 weeks later. Further details of the procedures during the initial session are provided in the following subsections.

#### Smartwatch Data Collection

At the initial study visit, 2 apps (Garmin and Labfront) were downloaded onto the participant’s smartphone to facilitate deidentified passive (raw sensor) and active data collection for the daily survey. The Garmin app facilitated data collection from the smartwatch sensors. The Labfront [33] app is a research platform that collects and organizes the Garmin smartwatch data and enabled the study team to monitor adherence and data collection in real time and remotely through a user-friendly study dashboard. The Labfront app was also used to deliver the daily EMA surveys.

A Garmin account was created for each participant using the Garmin Connect mobile app on their smartphone with deidentified information. The Labfront Companion app was then downloaded to the participant’s smartphone. The Labfront app uses a randomized 6-digit ID to connect the participant’s smartwatch to the app and to connect the Garmin and Labfront accounts. The Garmin smartwatch connects to the Garmin and Labfront apps via Bluetooth. Daily in-app Garmin and Labfront syncing required a Wi-Fi network or an LTE cellular data connection. Once the Garmin and Labfront apps were connected, data were available for real-time download by the study team in CSV format. Daily syncing of the apps enabled the aggregation of minute-by-minute data.

Participants were asked to wear the Garmin Vivosmart 4 smartwatch for 23 h/d on their nondominant wrist for 4 weeks and to charge it for approximately 1 h/d. Alarms were set in participant smartphones twice daily at times of the participant’s choosing to serve as reminders for the following: (1) charging the watch in the morning and (2) syncing the Garmin and Labfront apps and completing the daily survey on the Labfront app at night before going to sleep. To minimize the influence of external cues as well as behavior and health tracking information on participant behavior, all notifications were disabled on the study smartwatch, and additional features were removed from the Garmin app at the initial study visit.

Standardized training procedures with training criteria were used to teach participants how to charge the watch, sync the apps, and complete the daily survey. The training included demonstrations and a practice run during which participants were required to independently complete all daily study steps. Repetitions were recorded for practice runs, and the training session was timed. At the end of the training, participants were given a binder to take home and refer to during the study. The binder contained detailed instructions on how to charge the watch, sync the apps, and complete the daily survey during the study. The contents of this training binder are available in Multimedia Appendix 1.

#### 4-Week Monitoring Period

Participants were told to go about their daily life during the 4-week monitoring period while wearing the smartwatch for 23 h/d and to charge it for 1 h/d. Sleep duration, beat-to-beat heart rate variability, and physical activity data such as step count were collected but are not reported in this paper. Participants were also asked to complete the daily EMA survey delivered to their smartphones through the Labfront app. The EMA survey included 9 questions (Multimedia Appendix 1)*.* The purpose of the EMA survey was to guide the subsequent interpretation of smartwatch data (estimated time to complete the survey: 5 min).

During the course of the study, adherence was monitored through Labfront software by study personnel. If participants were nonadherent for >4 consecutive days, which was defined as wearing the watch for <16.67 h/d or not completing the daily surveys, they were contacted by the study team to determine the reason for nonadherence and to possibly reschedule the follow-up study session to extend the study period and obtain at least 28 days of data collection. The duration of the monitoring period (in days) was tallied for each participant. It is important to note that only 1 (2%) of the 47 enrolled participants required an extension of the monitoring period due to >4 consecutive days of missing data caused by a malfunction of their smartphone. Consequently, this participant was excluded from all feasibility and adherence analyses. For some of the participants (18/44, 41%), the monitoring extended beyond 4 weeks because the follow-up visit could not be scheduled on the study end date due to scheduling conflicts; in these cases, the follow-up visit was scheduled as soon as possible but no more than 1 week later.

#### Follow-Up Visit

After the study period, participants returned for a brief second visit (study visit 2) during which we collected data on their study experience, the usability of the apps and smartwatch, and barriers to future participation in wearable device research. At this visit, smartwatches were synced a final time to capture any aggregate-level data that had not been previously uploaded to the app. Subsequently, deidentified data obtained during the monitoring period were downloaded in CSV format for each participant from the Labfront researcher user interface for processing and analysis.

### Measures of Participant Characteristics

Cognitive tests and questionnaires used in standard clinical evaluations for cognitive decline were administered during study visit 1.

#### Cognition and Clinical Classification

IQ was estimated with the Hopkins Reading Test [34]. The 5 cognitive domains and 10 cognitive tests that were administered are listed in Table 1. Scores from each of the 10 tests were standardized (T score) after adjusting for demographic variables (age, education, sex, and estimated IQ score [28]). Clinical classification (ie, healthy cognition vs MCI) was based on published criteria [35] that define MCI as T scores of ≤40 (<1 SD) on both measures from at least 1 cognitive domain. A total composite score and cognitive domain–specific composite scores were calculated by averaging demographically corrected T scores (refer to Table 1 for all neuropsychological measures). These neuropsychological tests are used widely in research and clinic settings, have been extensively validated, and show strong psychometric properties [28].

#### Self-Reported Cognitive and Functional Decline

The average score from the Everyday Cognition (ECog)–short form [36] was used to estimate self-reported functional decline across a range of abilities (eg, memory and language), with scores ranging from 1 (no decline over the past 10 y) to 4 (worse decline over the past 10 y). The optimal cut score for distinguishing between healthy cognition and MCI is 1.32. The original validation study estimated a Cronbach α value of 0.96 [36].

### Smartwatch Feasibility and Adherence Measures

#### Training Time

The amount of time spent training (in minutes) during study visit 1 was recorded. Training procedures were standardized (Multimedia Appendix 1). Training began with a review of procedures for charging the watch and ended when the examiner observed that the participant independently demonstrated charging the watch, syncing the apps, and completing the daily survey.

#### Comprehension of Consent

Comprehension of consent was assessed using a 10-item quiz (Multimedia Appendix 1), which included questions on study-specific risks, data privacy, and security. The following is an example item: “The Garmin/Labfront apps collect the *content* of my texts and phone calls” (correct answer: no). To ensure informed consent, if participants answered any item incorrectly, study personnel immediately provided feedback to clarify the correct response.

#### Smartwatch Adherence

The total number of days that participants did not wear the watch was tallied, and 2 measures were used to evaluate adherence to the instruction to wear the watch for 23 h/d. First, the average daily wear time (in hours) per participant across all possible days of wear was collected. Wear time was derived from minute-to-minute heart rate data; therefore, wear time reflects the accumulation of heart rate data from the watch. Weekly averages of wear time were also computed (weeks 1, 2, 3, and 4) to examine adherence over time.

On the basis of published recommendations [37], daily watch data were considered valid if the participant wore the smartwatch for at least 16.67 hours that day. Therefore, watch adherence also was assessed as the “percentage of valid smartwatch days” during the monitoring period, calculated as the number of days the participants wore the watch for ≥16.67 h/d divided by the total number of days they were in the study.

#### EMA Survey Adherence

Adherence to the daily survey was calculated as the number of daily surveys completed divided by the total number of days that each participant was in the study. This percentage ranged from 0% to 100%, with 100% indicating perfect adherence.

### Usability Measures

A self-administered usability survey was completed by participants at the follow-up visit (study visit 2) to provide qualitative information on smartwatch use. The survey questions focused on the smartwatch and Labfront and Garmin smartphone apps, which were used for daily EMA surveys and syncing. Participants were also asked to report their likelihood of participating in another future study that included the smartwatch and daily surveys. This survey was administered to 45 (96%) of the 47 enrolled participants, including the participant whose monitoring period was extended.

### Statistical Analyses

Descriptive statistics were used to characterize feasibility: average daily wear time, study training time, percentage correct on consent comprehension, the percentage of completed EMA surveys, the percentage of valid smartwatch days, and responses to the study debriefing questionnaire. To examine univariate associations between feasibility and adherence measures and participant characteristics, Spearman correlations and Mann-Whitney *U* tests were used for continuous and categorical variables, respectively, with Bonferroni correction applied to interpret statistical significance. Friedman rank tests for 1-way repeated measures of ANOVA were used to analyze whether wear time differed from week 1 to week 4, with Bonferroni correction applied to interpret statistical significance. Nonparametric statistical tests were used due to the skewed distributions of the variables of interest. Multiple linear regressions were conducted to examine multivariate associations between demographics (ie, age, sex, race, and education) and cognitive variables (ie, ECog, cognitive composite scores, and cognitive status) on all feasibility and adherence measures. Separate models were conducted for each cognitive variable to avoid multicollinearity. As the dependent variables were not normally distributed, we used bootstrapping (5000 replications) to estimate robust SEs and CIs for coefficients, leading to more reliable testing of significance.

All analyses were conducted using SPSS software (version 28.0; IBM Corp) or, for bootstrapped regressions only, R (version 2023.12.1+402; R Foundation for Statistical Computing).

## Results

### Participant Characteristics

A total of 44 older adults ranging in age from 55 to 83 years participated and were included in the feasibility and adherence analyses. On average, participants were college educated with estimated IQ scores in the high average range (Table 2). Self-report of cognitive and functional decline was well within the normal range (ECog; range 1-2.5). The cognitive composite T score ranged from 35.40 to 63.40. The sample comprised mostly women (26/44, 59%) and primarily individuals identifying as Black or African American (11/44, 25%) or White, non-Hispanic (31/44, 70%). Of the 44 participants, 8 (18%) met the Jak-Bondi criteria for MCI. The participants were relatively active, with an average of 5999.74 (SD 2872.38) steps per day. Of the 44 participants, 17 (39%) owned a smartwatch before the study, with most owning a Fitbit device (n=9, 50%) or an Apple Watch (n=9, 50%). Those who currently or previously owned a smartwatch reported the main motivations for use as activity monitoring, followed by improving fitness and improving health. Of those who had not owned a smartwatch, most (17/44, 39%) indicated that they would consider buying one after their study experience.

**Table 2:** Participant characteristics and smartwatch feasibility measures (n=44).

Variables				Values
**Participant characteristics**				
	Age (y), mean (SD)			68.48 (7.22)
	Education (y), mean (SD)			16.41 (2.08)
	Sex: female, n (%)			26 (59)
	**Race and ethnicity, n (%)**			
		Asian	1 (2)	
		Black or African American	11 (25)	
		White, Hispanic	1 (2)	
		White, non-Hispanic	31 (70)	
	MCI^a^, n (%)			8 (18)
	Estimated IQ, mean (SD)			110.84 (7.59)
	ECog^b^ score, mean (SD)			1.41 (0.39)
	Cognitive composite score, mean (SD)			51.69 (5.68)
	Daily step count, mean (SD)			5999.74 (2872.38)
	Smartwatch ownership before study, n (%)			17 (39)
	“**If you already currently own and use a smartwatch or have previously, what is or was your main motivation for using the smartwatch? Select all that apply,” n (%)**			
		To monitor activities	15 (34)	
		Improve fitness	15 (34)	
		Improve health	12 (27)	
		Keep up with new technology	7 (16)	
		Improve appearance	2 (4)	
	“**If you do not already own a smartwatch, would you consider buying one after this experience?” n (%)**			
		Yes	17 (39)	
		No	10 (24)	
		Already have smartwatch	17 (39)	
**Smartwatch feasibility and adherence measures, mean (SD)**				
	Percentage correct of consent comprehension			98.00 (6.86)
	Average training time (min)			17.93 (6.89)
	Average daily smartwatch wear time (h)			21.04 (1.53)
	Percentage of valid smartwatch days			92.00 (10.39)
	Percentage of completed daily surveys			94.00 (9.58)

^a^MCI: mild cognitive impairment.

^b^ECog: Everyday Cognition.

### Retention Rate and Duration of Monitoring Period

A total of 47 participants were enrolled, of whom 1 (2%) dropped out after 1 day citing discomfort from the smartwatch band being too tight. All other participants (46/47, 98%) completed the study. However, 2 (4%) of these 46 participants were excluded from feasibility data analysis: 1 (50%) was excluded because their monitoring period was extended to 46 days due to a smartphone malfunction, and they did not complete the EMA survey for 1.5 weeks during the originally scheduled 28-day study; consequently, they were asked and agreed to extend their monitoring period. As a result, this participant was excluded from the analytic sample used for feasibility and adherence analyses. No other participants extended their monitoring period for reasons other than scheduling conflicts. Another participant was excluded due to mobility issues. Thus, the final analytic sample for the feasibility analyses comprised 44 participants.

The number of days monitored with the smartwatch ranged from 27 to 35. The mode for study days was 27 days, corresponding to exactly 4 weeks because the date of the initial study visit was excluded from analyses. The average number of days in the study was 29 (SD 3.46) days. Feasibility and adherence measures were computed using all available monitoring days.

### Feasibility and Adherence

Average feasibility and adherence measures are shown at the bottom of Table 2.

#### Comprehension Quiz

On average, participants obtained a score of 97% (range 70%-100%) correct on the consent comprehension quiz, approaching a perfect average score. The most frequently missed response was indicating “yes” to “Using the Garmin and Labfront apps will help improve my cognitive functioning.”

#### Training Time

Participants took an average of 17.93 (range 12-53) minutes to complete the study training during study visit 1. Training time was mostly spent independently practicing study tasks, such as syncing apps.

#### Smartwatch Adherence

Of the 44 participants, 11 (25%) did not wear the watch for at least 1 entire day (n=9, 20% missed only 1 d; n=1, 2% missed 2 d; and n=1, 2% missed 4 d). All participants wore the watch for ≥16.67 h/d for ≥14 days during the monitoring period. Of the 44 participants, 5 (11%) had ≥27 days of wear time that exceeded 16.67 h/d. On average, participants exceeded 16.67 h/d of wear time on 92% (range 53%-100%) of total study days. As the monitoring period extended beyond 4 weeks for some participants due to scheduling conflicts, watch adherence measures also were calculated over 27 days, with the average percentage of days exceeding 16.67 h/d of wear time remaining unchanged at 92% (range 55%-100%).

On average, participants wore the watch for 21 (range 15.71-22.44) h/d across the 4-week study period. Of the 44 participants, 3 (7%) had an average wear time of <18 h/d, 2 (5%) had an average wear time of <16.67 h/d, and 39 (89%) wore the watch for ≥20 h/d on average.

**Figure 1:**
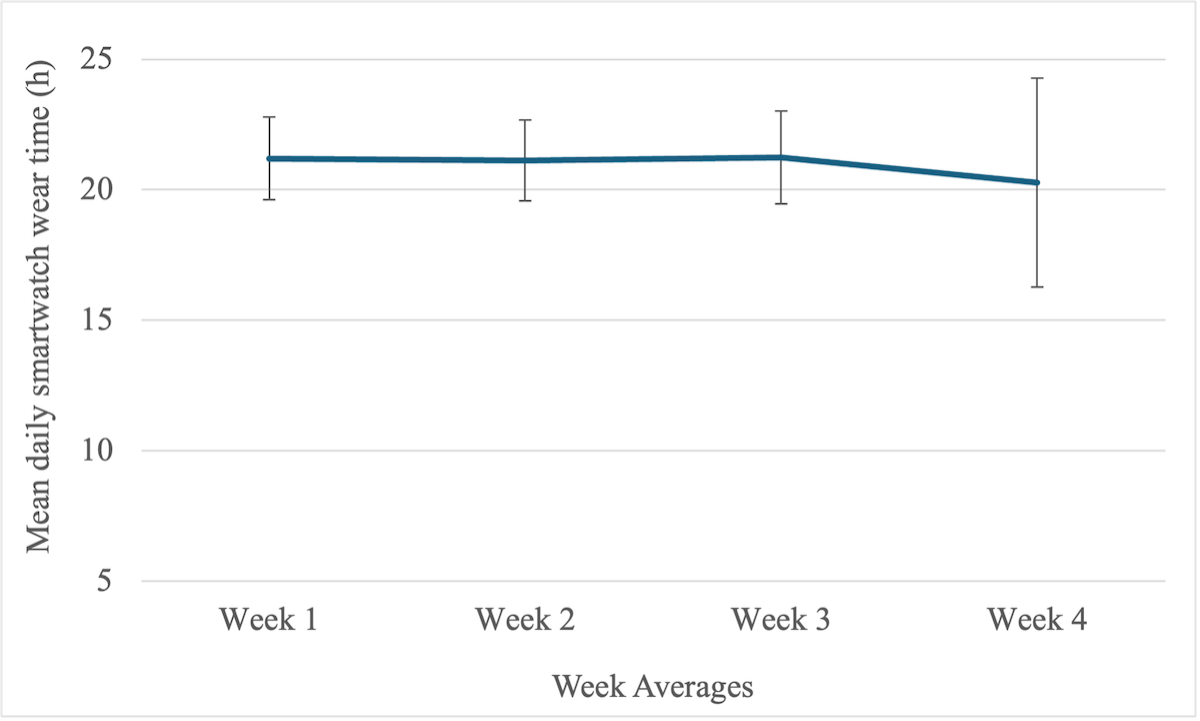
Daily wear time across participants was averaged across weeks 1 to 4 in the study. The y-axis represents the mean daily smartwatch wear time in hours. The error bars represent –1 to +1 SD.

The distribution of average watch wear time per week is shown in Figure 1. Pairwise 2-tailed *t* tests showed no significant difference in average wear time across the 4 weeks (refer to Multimedia Appendix 1 for full results).

**Figure 2:**
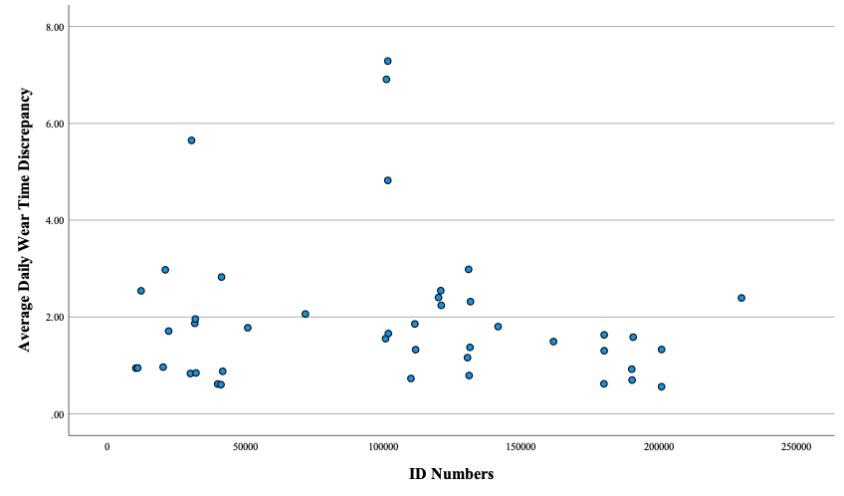
Average wear time discrepancy for the entire sample. The y-axis represents each participant’s average daily wear time subtracted from the required 23 h/d. The x-axis shows participant ID numbers from the feasibility analyses. Each dot represents a participant.

As shown in Figure 2, on average, 91% (40/44) of the participants wore the watch for at least 19 h/d, within 4 hours of the required 23 h/d. In fact, on average, participants on average wore the watch for 21 h/d, within 2 hours of the required 23 h/d.

#### EMA Survey Adherence

On average, participants completed surveys on most study days (94%; range 58%-100%). Of the 44 participants, 3 (7%) completed <70% of the surveys, while 41 (93%) completed >85% of the daily surveys.

### Correlations Among Smartwatch Feasibility and Adherence Metrics

As shown in Table 3, a greater percentage of daily surveys completed was significantly associated with longer study training time, but the *P* value for this association did not survive Bonferroni correction (ie, *P*=.005 and below). Correlations between the 2 measures of smartwatch adherence and between smartwatch adherence measures and the percentage of daily surveys completed were significant and survived correction for multiple comparisons. The correlations indicated that participants who completed a higher percentage of daily surveys also wore the watch for longer durations and had more days of valid smartwatch wear time.

**Table 3:** Correlation coefficients among smartwatch feasibility and adherence metrics.

Variables		Consent comprehension percentage correct	Study training time	Percentage of daily surveys completed	Percentage of valid smartwatch wear days
**Study training time**					
	*r*	0.055	—^a^	—	—
	*P* value	.77	—	—	—
**Percentage of daily surveys completed**					
	*r*	–0.016	0.298	—	—
	*P* value	.92	.049	—	—
**Percentage of valid smartwatch wear days**					
	*r*	0.230	0.018	0.544	—
	*P* value	.13	.91	<.001	—
**Daily wear time**					
	*r*	0.095	0.091	0.579	0.778
	*P* value	.54	.56	<.001	<.001

^a^Not applicable.

### Associations Between Participant Characteristics and Feasibility and Adherence Metrics

As shown in Table 4, only age and self-reported cognitive and functional decline (ECog) were significantly associated with feasibility and adherence metrics. Specifically, older participants required longer study training times, and participants who reported greater cognitive and functional decline (ECog) had lower daily average wear time. However, these significant correlations did not survive Bonferroni correction (*P*=.003). No other correlation coefficients were statistically significant (Table 4).

**Table 4:** Correlation coefficients between smartwatch feasibility metrics and demographic and clinical factors.

Variables			Consent comprehension percentage correct		Study training time		Percentage of daily surveys completed		Percentage of valid smartwatch wear days		Daily wear time
**Age**											
	*r*	–0.164		0.403		0.165		–0.023		0.006	
	*P* value	.29		.007		.28		.88		.97	
**Education**											
	*r*	0.041		0.029		–0.197		–0.092		–0.108	
	*P* value	.79		.85		.20		.55		.48	
**Cognitive composite scores**											
	*r*	0.216		–0.002		0.037		0.102		0.155	
	*P* value	.16		.99		.81		.51		.31	
**ECog^a^**											
	*r*	–0.102		–0.206		–0.221		–0.261		–0.393	
	*P* value	.51		.19		.15		.09		.009	

^a^ECog: Everyday Cognition.

There were no significant sex differences in consent comprehension, training time, watch adherence measures, or the percentage of daily surveys completed (data reported in Multimedia Appendix 1). Participants with MCI and those with healthy cognition differed on only 1 measure (data reported in Multimedia Appendix 1): participants with MCI (median 20.64, IQR 2.74 h) had significantly lower daily wear time than participants with healthy cognition (median 21.53, IQR 1.18 h; *U*=71.00; *P*=.03). This result is consistent with the significant correlation between ECog and daily wear time reported in Table 4, suggesting an association between lower cognitive abilities and less smartwatch wear time.

Between-group analyses comparing Black and White participants were not statistically significant, except for daily wear time (data reported in Multimedia Appendix 1). Black participants (median 20.76, IQR 0.91 h) had significantly lower daily smartwatch wear time than White participants (median 21.83, IQR 1.02 h; *U*=74.00; *P*=.004).

Multivariate associations between participant characteristics and feasibility and adherence metrics were investigated using multiple regression analyses with bootstrapping to estimate the significance of the coefficients. Separate regressions were run, including one for each cognitive variable (cognitive composite scores, ECog, and cognitive status; refer to Multimedia Appendix 1 for all tables), to avoid multicollinearity. The results showed that race remained a significant predictor of daily wear time, even after controlling for age, sex, education, and measures of cognition, with White participants having longer daily wear times than Black participants. Multivariate analyses also showed that age was significantly associated with training time even after controlling for sex, education, race, and measures of cognition, with older age associated with longer study training time. By contrast, associations between the measures of cognition and feasibility and adherence metrics were not statistically significant in regression analyses, including demographic variables (age, sex, education, and race).

### Usability Survey

The results of the usability survey are shown in Table 5 (Garmin smartwatch) and Table 6 (Labfront smartphone app). Overall, participants expressed favorable views of both the smartwatch and the smartphone apps and especially enjoyed their involvement in research. When asked, “Would you wear this Garmin smartwatch without being asked to wear it as part of the study?” 55% (25/45) of the sample reported “yes.” However, all participants indicated they were likely (11/45, 24%) or very likely (34/45, 76%) to participate in a future study asking them to wear a Garmin smartwatch daily. Of the 45 participants, 36 (80%) either somewhat or strongly agreed that they had a positive experience using the Garmin smartwatch during the study period (Table 5). Most of the participants (31/45, 68%) reported that the smartwatch was comfortable or very comfortable. Most of the participants (32/45, 71%) were satisfied or very satisfied with the functioning of the watch. The majority of the complaints about the smartwatch were regarding low battery life (17/45, 38%), followed by discomfort (8/45, 18%) and technical issues (8/45, 18%). Of the 45 participants, 17 (38%) reported no complaints about the smartwatch. Participants suggested the following improvements for the watch: changing wristband material (18/45, 40%), increasing the display size (15/45, 33%), and increasing the wristband size (10/45, 22%).

**Table 5:** Smartwatch usability and satisfaction (n=45).

Survey items			Participants, n (%)
“**How comfortable was the smartwatch?”**			
	*Very comfortable^a^*	20 (44)	
	Comfortable	11 (24)	
	Somewhat comfortable	10 (22)	
	Not comfortable	4 (9)	
“**Did you wear it overnight every night?”**			
	Yes	44 (98)	
“**How satisfied were you with the functioning of the watch (i.e. you were able to tell date/time easily)?”**			
	*Very satisfied*	18 (40)	
	Satisfied	14 (31)	
	Somewhat satisfied	9 (20)	
	Not satisfied	4 (9)	
“**Do you have any complaints about using the Garmin Vivosmart 4 watch?”**			
	*None*	17 (38)	
	Technical issues	8 (18)	
	Doesn’t fit	2 (4)	
	It’s uncomfortable	8 (18)	
	Low battery life	17 (38)	
	Problems with the screen	6 (13)	
“**Overall, I had a positive experience using the smartwatch.”**			
	Disagree strongly	0 (0)	
	Somewhat disagree	3 (7)	
	Neutral	5 (11)	
	Somewhat agree	18 (39)	
	*Agree strongly*	19 (43)	
“**What would you change to improve the comfort of wearing the Garmin smartwatch?”**			
	No changes needed	15 (33)	
	Improve wristband clasp function	9 (20)	
	Increase display size	15 (33)	
	*Change material of wristband (cloth, material)*	18 (40)	
	Other (increase size of wristband)	10 (22)	
“**How satisfied were you with charging the battery of the Garmin smartwatch?”**			
	Very satisfied	10 (22)	
	*Satisfied*	19 (44)	
	Somewhat satisfied	11 (24)	
	Not satisfied	4 (9)	
“**Did the watch ever run out of battery while you were wearing it?”**			
	Yes (those who answered yes, indicated between 1 and 5 times)	17 (38)	

^a^Italicized text indicates the most frequent response.

App usability and overall experience are reported in Table 6. Of the 45 participants, 41 (91%) were satisfied or very satisfied with the syncing process of both apps (Garmin and Labfront). Of the 45 participants, 35 (78%) indicated that they agreed or strongly agreed that the Labfront app was easy to use and that they felt very confident using it. Importantly, 64% (29/45) of the participants had a positive or extremely positive experience with the Labfront app. Moreover, 71% (32/45) of the participants indicated that the Labfront app was easy to use or extremely easy to use (Table 6).

**Table 6:** App usability and overall experience (n=45).

Survey items			Participants, n (%)
“**How easy was it to sync the watch every day with the Labfront and Garmin apps?”**			
	*Very satisfied^a^*	32 (71)	
	Satisfied	9 (20)	
	Somewhat satisfied	1 (2)	
	Not satisfied (issues included having to try a couple of times to sync the Labfront app)	3 (7)	
“**I thought Labfront was easy to use”**			
	Strongly disagree	3 (7)	
	Disagree	5 (11)	
	Neither agree nor disagree	2 (4)	
	*Agree*	18 (40)	
	Strongly agree	17 (37)	
“**I felt very confident using the Labfront app”**			
	Strongly disagree	2 (4)	
	Disagree	2 (4)	
	Neither agree nor disagree	4 (9)	
	Agree	18 (40)	
	*Strongly agree*	19 (42)	
“**I enjoy using the Labfront app”**			
	Strongly disagree	1 (2)	
	Disagree	2 (4)	
	*Neither agree nor disagree*	18 (40)	
	Agree	17 (38)	
	Strongly agree	7 (16)	
“**How would you rate your experience with the Labfront app?”**			
	Extremely negative	1 (2)	
	Negative	2 (4)	
	Neutral	13 (29)	
	*Positive*	23 (51)	
	Extremely positive	6 (13)	
“**How would you rate usability of the Labfront app?”**			
	Very difficult to use	0 (0)	
	Somewhat difficult to use	3 (7)	
	Neutral	10 (22)	
	*Easy to use*	23 (53)	
	Extremely easy to use	8 (18)	

^a^Italicized text indicates the most frequent response.

## Discussion

### Principal Findings

Overall, the results demonstrated that our longitudinal study requiring daily wear of a smartwatch and daily EMA survey completion in a racially diverse sample of older adults was feasible with excellent adherence. Participants with cognitive impairment and those without wore a commercially available smartwatch during waking and sleep hours for 4 weeks for an average of 21 h/d and showed an average response rate of 94% to daily EMA surveys. Only 1 (2%) of the 47 enrolled participants refused to complete the study after informed consent due to discomfort with the watch fit. On the basis of the results of our consent comprehension quiz, participants had no difficulty understanding the study procedures and risks, and completed smartwatch and app training in 18 minutes on average. After consent and training, participants remained in the study for at least 28 days. Average wear time did not decline significantly over the course of the study and on average never fell below 20 h/d. Although there are no benchmark standards for indicating good smartwatch adherence, a recent systematic review of activity trackers to monitor physical activity suggested that 3 valid days of at least 10 h/d may be a good adherence threshold for a week-long study [38]. Another study defined a “valid day” as one that includes at least 16.67 hours of data or at least 600 one-minute epochs of nonzero heart rate values [37]. In our study, participants wore the study watch for >16.67 h/d on 92% of the days on average. Thus, our results exceed the recent suggestions for adherence and validity and show strong support for the feasibility and adherence of our protocol to monitor older adults.

Smartwatch feasibility and adherence metrics were associated with each other as expected, and EMA survey adherence also was related to smartwatch adherence measures. However, feasibility and adherence metrics were differentially related to participant factors, indicating the importance of measuring adherence to each feature of a smartwatch and EMA study separately. EMA survey adherence was not related to any participant characteristic and was uniformly high, possibly because participants received smartphone reminder alarms to complete the daily survey and sync the apps at predetermined times at night before bedtime. There was no comparable alert to prompt participants to wear the smartwatch during the day or night. Future studies should consider adding prompts to facilitate smartwatch adherence.

In contrast to past studies [21], we observed no associations between participant’s sex or education level on any measure of adherence and feasibility in our diverse sample. Smartwatch adherence was associated with race such that White participants wore the smartwatch for more hours per day (daily wear time) than Black participants during the study period. The effect of race remained significant, even after controlling for other demographic variables, including age, education, and cognition. The reason for the race difference is unknown but may be explained by different beliefs, attitudes, or daily habits between the racial groups. As wear time is measured using the photoplethysmography sensor, which relies on the absorption of infrared light into the skin, it is possible that the measures might have been less accurate for people with darker skin tone. If replicated, cultural factors and potential sensor limitations related to skin tone, which might explain racial differences in adherence, should be systematically explored. Although Black and White participants differed in daily wear time, race did not influence the percentage of days that reached the 16.67-hour threshold for “valid” wear time. Thus, the race difference in daily wear time was small and potentially inconsequential. Furthermore, race was not associated with any other feasibility and adherence measure.

Although cognitive ability level (both self-reported cognitive decline and MCI status) was associated with daily wear time in bivariate analyses, the effect of cognition was not significant in multiple regression models. These results contrast with past research that has shown cognitive ability to be related to adherence; for example, memory ability was related to adherence in a study requiring the daily syncing of a Fitbit device in a sample of older adults without cognitive impairment [21]. Our study protocol, including training and supports during the monitoring period (eg, the take-home binder and twice daily alarms [in the morning to charge the watch and at night to sync the apps and complete the surveys]) may have helped participants with cognitive difficulties adhere to the smartwatch wear requirements and EMA survey.

Age was not associated with watch or survey adherence, which is consistent with the results from a recent smartphone digital phenotyping study [15]. However, similar to a recent study from our laboratory [13], we found that age was associated with training time such that older participants required longer training times in the laboratory to learn how to sync the study-related apps and complete the daily EMA survey on their smartphone. The training required participants to independently complete each activity (syncing apps, charging the smartwatch, and completing the EMA survey) while a member of the study team observed, answered questions, and provided feedback. On average, participants took <20 minutes to complete the training. Older participants may have taken longer to complete the training due to lower digital literacy; they may have required more practice and may have asked more questions during the training. Although the number of people aged >65 years owning smartphones in the United States has steadily increased over the past decade, digital literacy is generally lower in older adults. The longer training time and the take-home binder, which summarized the training for review during the study period, may have consequently minimized age effects on smartwatch and daily EMA survey adherence. Smartwatch studies that incorporate a “human in the loop” have been found to have higher adherence than fully remote studies [15]. Even if a study is conducted fully remotely, providing some contact with study personnel (via teleconference) or training resources (in the form of a training binder, video tutorial, website, or web-based helpline) could be quite beneficial to increasing adherence, especially for older adults with low digital literacy.

Participants generally demonstrated solid knowledge of the informed consent information, including issues related to data security and privacy that are relevant to research using wearable devices; for example, responses to the study consent quiz showed that participants understood that the study apps were Health Insurance Portability and Accountability Act–compliant and that they had control over their data such that they had the right to delete their study data at any time. In contrast to a study on smartphone digital phenotyping that found that education and race were associated with quiz accuracy [13], percentage correct on the consent comprehension quiz was not significantly related to any of the participant demographic factors, possibly because the scores on the quiz had little variability (ie, ceiling effects). Similar to a past study [13], the most frequently incorrect question concerned the potential benefits of the study, with many participants incorrectly reporting that their participation would improve their cognitive abilities. In these cases, the comprehension quiz enabled the study team to explain that wearing the smartwatch had no clear benefit and possibly preclude participants’ disappointment at the end of the study.

Smartwatch usability reports were largely very positive, with most of the participants (37/45, 82%) agreeing that they had a positive experience using the Garmin Vivosmart 4 smartwatch in the study as well as high satisfaction regarding its comfort and functioning. This is in line with past research showing that the Garmin Vivosmart was rated as the most usable and acceptable smartwatch compared to 5 other smartwatches by older adults in a small study [39]. Although participants indicated that they needed to charge the Garmin Vivosmart 4 smartwatch daily, they were generally satisfied with battery life. Regarding app usability, almost all participants were satisfied with the syncing process of the Labfront and Garmin Connect apps (42/45, 93%), felt that the Labfront app was easy to use (35/45, 78%), and reported a positive experience using the Labfront app (29/45, 64%). There are currently no other studies examining the usability and acceptability of the Labfront and Garmin Connect apps.

Regarding the usability of the smartwatch and apps, participants were generally satisfied with the smartwatch and reported that they would wear it again in another study. It is important to consider that most of the participants (44/45, 98%) reported having to charge the watch daily, and low battery life was the most frequently cited complaint about the smartwatch. Thus, in future studies, participants should be trained and prompted to charge watches and other digital devices, and they should be informed about battery limitations to preclude complaints and dissatisfaction. Multiple chargers, including portable batteries, may be offered to participants to improve usability. Despite minor complaints regarding charging, most of the participants (37/45, 82%) reported a positive experience during the study using the smartwatch. Participants also reported high satisfaction and ease of use of the Labfront and Garmin apps, which were used for daily syncing to enable sensor and EMA data to be transmitted to the research team. Taken together, our findings are generally in line with prior research showing good acceptability and usability of wearable devices in research studies involving older adults [21].

### Limitations

Although our sample included older adults of different races, the sample was relatively small and highly educated. Furthermore, only 8 (18%) of the 44 participants had MCI; therefore, analyses comparing participants with MCI and those with healthy cognition were underpowered. However, cognitive effects on adherence were also examined with a composite test score and self-reports of cognitive and functional abilities (ECog). Moreover, our monitoring period was limited to a 4-week period; therefore, the conclusions may not generalize to longer periods. Importantly, our study incorporated several supports for participants such as in-person training, a take-home binder, contact with study personnel, and reminder alarms set for times specified by participants. These study features likely influenced the high adherence to smartwatch wear and EMA survey completion in our study compared to fully remote studies with little to no support from study personnel. In addition, our study’s EMA survey imposed minimal demands because it was required only once daily and completed at a time preferred by participants.

### Conclusions

This study demonstrated the feasibility of a combined smartwatch monitoring and daily EMA study requiring nearly 24-hour wear time daily for a month in racially diverse older adults, including those with cognitive impairment and those without. Smartwatch feasibility and adherence metrics were not significantly intercorrelated and were differentially related to participant characteristics. The inclusion of a guided training for older adult participants on study procedures as well as a take-home binder and smartphone reminder alarms to complete study steps (syncing apps, charging the smartwatch, and completing the daily EMA survey) may have contributed to strong adherence and feasibility in this study, regardless of cognitive impairment. Participants reported high satisfaction regarding the usability of both the Garmin Vivosmart 4 smartwatch and Labfront app. Overall, longitudinal monitoring using a commercially available smartwatch with a daily EMA survey is acceptable and feasible for collecting nearly continuous data in Black and White older adults, including those with cognitive impairment and those without.

## Appendix

Multimedia Appendix 1 Training binder, consent comprehension quiz, and supplementary analyses.

## Abbreviations

AD: Alzheimer disease
ADRD: Alzheimer disease and related dementias
ECog: Everyday Cognition
EMA: ecological momentary assessment
MCI: mild cognitive impairment
