# Supplementary material for: Longitudinal and Combined Smartwatch and Ecological Momentary Assessment in Racially Diverse Older Adults: Feasibility, Adherence, and Acceptability Study
Source: JMIR Hum Factors. 2025 Apr 8;12:e69952. doi: 10.2196/69952 (PMC12015335; doi:10.2196/69952)

Supplement

Comprehension of Consent Quiz

**1.** This study requires downloading the the Labfront and Garmin apps which collects information from my smartwatch sensors and daily survey responses	Yes	No
**2.** Using the Garmin and Labfront apps will help improve my cognitive functioning	Yes	No
**3.** The Garmin/Labfront apps collects the *content* of my texts and phone calls	Yes	No
**4.** The Labfront/Garmin apps use a secure encryption system to make information that it collects unidentifiable and untraceable	Yes	No
**5.** The Garmin/Labfront apps and its data collection procedures are HIPAA compliant	Yes	No
**6.** The information collected from my smartwatch by Garmin/Labfront may be sold, traded or transferred to other outside parties	Yes	No
**7.** The de-identified information collected by Labfront/Garmin is linked to me using a random ID; only the study staff have the key linking me to this ID	Yes	No
**8.** It is possible that I may experience some discomfort wearing the watch up to 23 hours in a day	Yes	No
**9.** I can ask the study staff to delete all of my information collected by Garmin/Labfront at any time	Yes	No
**10.** The server that stores your Garmin/Labfront study data is maintained in a secured facility behind a firewall	Yes	No

EMA Survey Questions

1. How typical was your day today on a scale of 1 (very typical) to 5 (very atypical)?

1 – very typical; I spent today exactly how I spend most days
2 – typical; for the most part, today was similar to how I spend most days
3 – somewhat typical; I spent my day doing a few things I don't usually do
4 – atypical; today was not a typical day for me
5 – very atypical; today was extremely different from most days

2. Were there any changes to the medications you usually take, or to your overall health today?
Yes
No

3. If yes, please explain briefly

4. How long did it take you to charge and sync the watch today (in minutes)?

5. How sharp did you feel today (in terms of your thinking skills, like your memory, concentration, and speaking abilities)?
Required
Very sharp; my mind was better than usual!
Neutral; my mind felt like it normally does
Not sharp; my mind felt slower than usual

6. How was your mood today?
Required
Happy
Neutral
Sad

7. How stressed did you feel today on a scale from 1 (not stressed at all) to 10 (most stressed)?

8. How long did you exercise today if at all (in minutes)?

9. How many hours of sleep did you get the night before approximately?

10. How much time did you spent socializing today (i.e., interacting with other people) in minutes?

Analyses Examining Differences in Average Wear Time Across Weeks 1-4
Pairwise comparisons examining the differences in the weekly averages of wear time shown in Figure 2 of the paper are reported in the table below. All comparisons were not statistically significant, indicating no differences in average wear time across the study weeks.

Pairwise Comparisons	Test Statistic	Std. Error	Std. Test Statistic	p
Week 2 vs. Week 4	-.148	.275	-.537	.591
Week 2 vs. Week 3	-.167	.275	.784	.433
Week 2 vs. Week 1	.450	.275	.826	.409
Week 4 vs. Week 3	.089	.275	.248	.804
Week 4 vs.Week 1	.122	.275	.289	.773
Week 3 vs. Week 1	.011	.275	.041	.967

Differences between Men and Women on Feasibility Metrics

As shown in the table below, there were no statistically significant differences between men (n=18) and women (n = 26) on any of the feasibility/adherence measures.

	**Women**		**Men**		**Mann Whitney U Test**		
	Median	IQR	Median	IQR	U	Z	P
**Comprehension of Consent Quiz**	100	0	100	0	223.00	-.440	.660
**Percent of Daily Surveys Completed**	.98	.07	.98	.09	231.00	-.077	.939
**Daily Wear Time**	21.37	1.28	21.43	1.70	289.00	1.313	.189
**Study Training Time**	16	6	17	5	233.00	-.024	.981
**Percent of Valid Smartwatch Days**	.96	.06	.96	.10	272.50	.932	.352

Differences between Participants with Mild Cognitive Impairment and Healthy Cognition on Feasibility Metrics

As shown in the table below, participants with healthy cognition (HC, n = 36) versus mild cognitive impairment (MCI, n =8) differed significantly on daily wear time, with participants with healthy cognition wearing the watch for more hours per day. No other feasibility/adherence measures were different between the MCI and HC groups.

	HC		MCI		Mann Whitney U Test		
	Median	IQR	Median	IQR	U	Z	P
**Comprehension of Consent Quiz**	100	0	100	15	123.00	-1.072	.540
**Percent of Daily Surveys Completed**	1.00	.06	.97	.12	105.50	-1.253	.247
**Daily Wear Time**	21.53	1.18	20.64	2.74	71.00	-2.221	.025
**Study Training Time**	17	6	15	8	115.50	-.873	.393
**Percent of Valid Smartwatch Days**	.96	.07	.91	.21	94.00	-1.542	.134

Race Differences on Feasibility Metrics

There were insufficient numbers of participants to do a full comparison of all race/ethnic groups. Therefore, only differences between participants who identified as Black race (n =11) versus White race (n=31) were analyzed. As shown in the table below, only the difference in daily wear time was significantly different between the groups, with White participants wearing the watch more often during the day. There were no other significant differences between the race groups.

	White Race		Black Race		Mann Whitney U Test		
	Median	IQR	Median	IQR	U	Z	P
**Comprehension of Consent Quiz**	100	0	100	0	172.00	-.432	.666
**Percent of Daily Surveys Completed**	100	5	96.77	15	137.00	-1.290	.197
**Daily Wear Time**	21.67	1.12	20.76	1.21	74.00	-2.914	.004
**Study Training Time**	16	6	16	5	175.50	-.164	.870
**Percent of Valid Smartwatch Days**	96.30	7	90.32	9	122.500	-1.621	.105

Bootstrapped Linear Regression Analyses Predicting Each Adherence/Feasibility Metric with Ecog

Each regression model includes age, sex, education race, and each cognitive variable separated (Ecog, cognitive composite, and cognitive status) into different models.

Parameter	Statistic	Consent Comprehension	Study Training Time	Percent of Daily Surveys Completed	Percent of Valid Smartwatch Wear Days	Daily Wear Time
(Intercept)	Coefficient	102.69	4.7	0.884	1.103	24.337
	CI	[75.06, 125.5]	[-15.06, 27.77]	[0.58, 1.15]	[0.83, 1.38]	[20.32, 28.48]
	p	0	0.65	0	0	0
Sex (1=Male)	Coefficient	-0.175	-3.228	-0.024	-0.006	0
	CI	[-4.2, 4.01]	[-9.05, 0.66]	[-0.09, 0.03]	[-0.09, 0.06]	[-1.12, 0.89]
	p	0.928	0.12	0.411	0.844	1
Education	Coefficient	0.155	-0.183	-0.008	-0.005	-0.082
	CI	[-0.65, 0.93]	[-1.06, 0.53]	[-0.02, 0]	[-0.02, 0.01]	[-0.35, 0.09]
	p	0.677	0.595	0.166	0.446	0.373
Age	Coefficient	-0.08	0.293	0.003	0	-0.001
	CI	[-0.37, 0.28]	[0.06, 0.57]	[0, 0.01]	[0, 0]	[-0.05, 0.05]
	p	0.619	**0.009**	0.059	0.898	0.965
Race (1=Black)	Coefficient	-0.281	-1.612	-0.061	-0.061	-1.145
	CI	[-5.99, 6.69]	[-5.96, 1.25]	[-0.15, 0.01]	[-0.17, 0.02]	[-2.71, -0.15]
	p	0.926	0.292	0.092	0.154	**0.023**
Ecog	Coefficient	-1.139	-1.429	-0.005	-0.05	-1.074
	CI	[-8.85, 3.68]	[-7.22, 3.11]	[-0.11, 0.07]	[-0.13, 0.05]	[-2.13, 0.41]
	p	0.66	0.545	0.917	0.332	0.157

Bootstrapped Linear Regression Analyses Predicting Each Adherence/Feasibility Metric with Cognitive Composite

Parameter	Statistic	Consent Comprehension	Study Training Time	Percent of Daily Surveys Completed	Percent of Valid Smartwatch Wear Days	Daily Wear Time
(Intercept)	Coefficient	86.879	8.254	0.847	0.955	21.907
	CI	[41.8, 117.3]	[-10.26, 29.22]	[0.37, 1.34]	[0.57, 1.41]	[16.56, 29.51]
	p	0.001	0.359	0	0	0
Sex	Coefficient	-0.127	-3.106	-0.026	-0.002	0.091
	CI	[-3.89, 4.04]	[-8.33, 0.67]	[-0.09, 0.03]	[-0.08, 0.06]	[-1.03, 0.96]
	p	0.94	0.12	0.384	0.951	0.863

Education	Coefficient	0.135	-0.24	-0.008	-0.006	-0.108
	CI	[-0.66, 0.86]	[-1.16, 0.51]	[-0.02, 0]	[-0.02, 0.01]	[-0.39, 0.08]
	p	0.675	0.535	0.14	0.334	0.266
Age	Coefficient	-0.165	0.372	0.003	0	0
	CI	[-0.48, 0.18]	[0.13, 0.68]	[0, 0.01]	[0, 0]	[-0.05, 0.06]
	p	0.314	**0.002**	0.098	0.853	0.992
Race	Coefficient	1.415	-2.385	-0.057	-0.055	-1.144
	CI	[-4.92, 8.64]	[-7.8, 0.89]	[-0.15, 0.01]	[-0.17, 0.03]	[-2.88, -0.18]
	p	0.674	0.193	0.08	0.2	**0.016**
Cognitive Composite	Coefficient	0.404	-0.184	0.001	0.002	0.023
	CI	[-0.05, 0.92]	[-0.54, 0.06]	[-0.01, 0.01]	[0, 0.01]	[-0.07, 0.09]
	p	0.097	0.146	0.766	0.484	0.549

Bootstrapped Linear Regression Analyses Predicting Each Adherence/Feasibility Metric with Cognitive Status

Parameter	Statistic	Consent Comprehension	Study Training Time	Percent of Daily Surveys Completed	Percent of Valid Smartwatch Wear Days	Daily Wear Time
(Intercept)	Coefficient	98.809	-0.054	0.85	0.988	22.118
	CI	[66.78, 124.32]	[-19.09, 16.01]	[0.49, 1.19]	[0.69, 1.3]	[17.95, 27.08]
	p	0	0.993	0	0	0
Sex	Coefficient	-0.131	-3.107	-0.024	0.001	0.097
	CI	[-3.94, 4.01]	[-8.71, 0.73]	[-0.09, 0.03]	[-0.07, 0.06]	[-1.04, 0.98]
	p	0.937	0.13	0.404	0.982	0.865
Education	Coefficient	0.224	-0.157	-0.007	-0.004	-0.08
	CI	[-0.5, 1.09]	[-1.06, 0.57]	[-0.02, 0.01]	[-0.02, 0.01]	[-0.35, 0.15]
	p	0.518	0.679	0.303	0.618	0.495
Age	Coefficient	-0.065	0.329	0.003	0	0.009
	CI	[-0.36, 0.32]	[0.11, 0.64]	[0, 0.01]	[0, 0]	[-0.04, 0.06]
	p	0.703	**0.002**	0.081	0.834	0.702
Race	Coefficient	0	-1.178	-0.057	-0.059	-1.183

	CI	[-5.67, 6.49]	[-5.3, 1.49]	[-0.14, 0.01]	[-0.17, 0.03]	[-2.72, -0.09]
	p	0.999	0.434	0.097	0.194	**0.034**
**MCI**	Coefficient	-3.312	-1.912	-0.035	-0.057	-0.842
	CI	[-11.33, 3.92]	[-5.98, 2.04]	[-0.14, 0.06]	[-0.15, 0.06]	[-2.08, 0.6]
	p	0.391	0.319	0.481	0.295	0.242

Using the Labfront/ Garmin Apps

Participant Training Procedures

Examiner: record start time
(& whether in-person or via zoom)

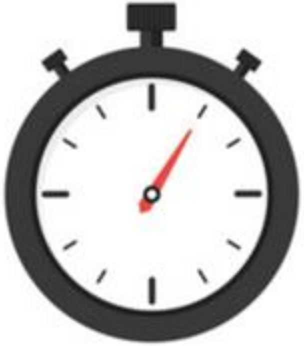

Basic Functions

The Garmin and Labfront apps are designed to:

1. Collect de-identified data from your watch sensors throughout the day without any interference while you go about your regular daily activities.

2. Provide you with a brief 9-question survey on your phone via the Labfront app. The survey should take about **5 minutes** to complete.

This is what the apps will look like on your phone screen.

(show on participant's phone)

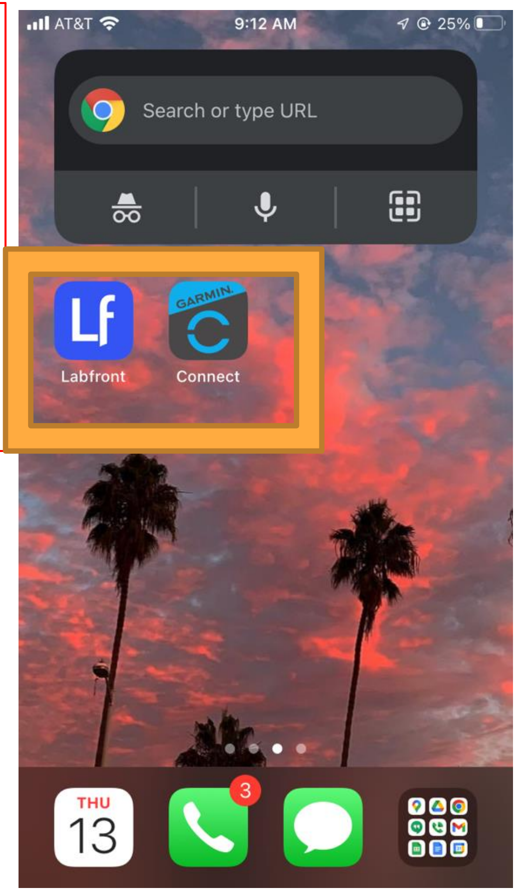

Reviewing Tasks

1. Charging the watch
2. Syncing the apps
3. Completing the daily survey

In the next few slides, we will show you how to do each on your phone.

You will receive an alarm twice per day, one to charge your watch in the morning, and one to complete the survey + sync the apps at night.

Your morning survey alarm to charge the watch is scheduled for ________ am

Your nightly survey alarm to complete the survey/sync apps is scheduled for ________ pm

Task #1: Charging the Watch

Morning

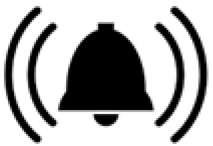

STEP 1:
Alert will appear

You will hear or see an alert on your phone...
Click “Stop”

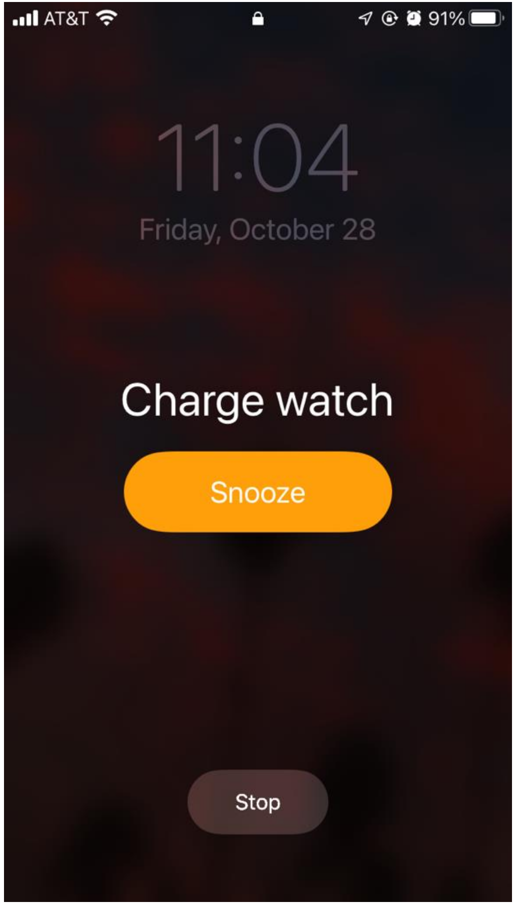

If you are using your phone when you get an alert, the notification will look like this...

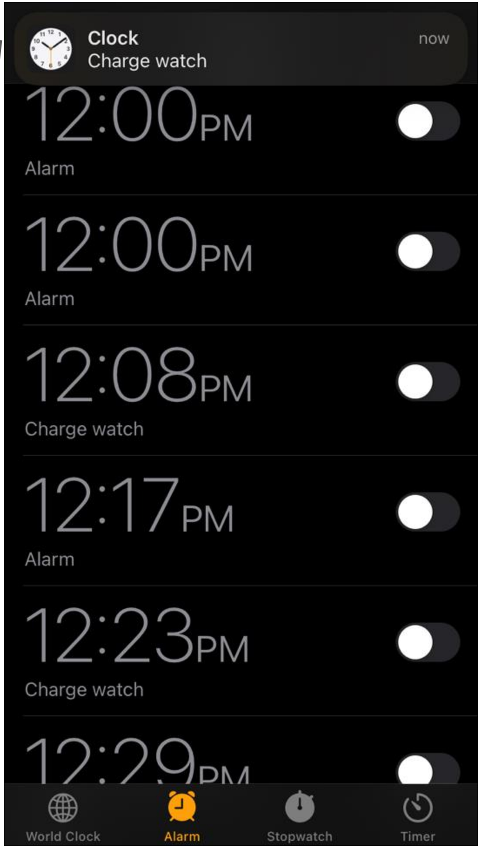

Task #1- how to charge the watch

- Please try to make sure your watch battery does not die during the study!
- You can plug the cord into a wall outlet to charge in the wall!
- Plan on charging it for about An hour every day every morning

To prevent corrosion, thoroughly clean and dry the contacts and the surrounding area before charging or connecting to a computer. Refer to the cleaning instructions in the appendix.

1. Align the charger posts with the contacts on the back of the device, and press the charger (1) until it clicks.

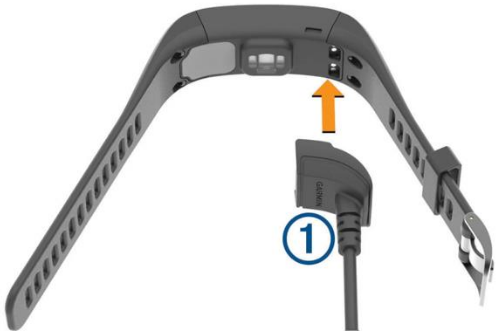

2. Plug the USB cable into a USB port on your computer.
3. Charge the device completely.

Charging the Watch and Completing Task

- Open the Labfront app, Click on the “Charge your watch” task after you have charged it
- Then click ”Mark as Complete”

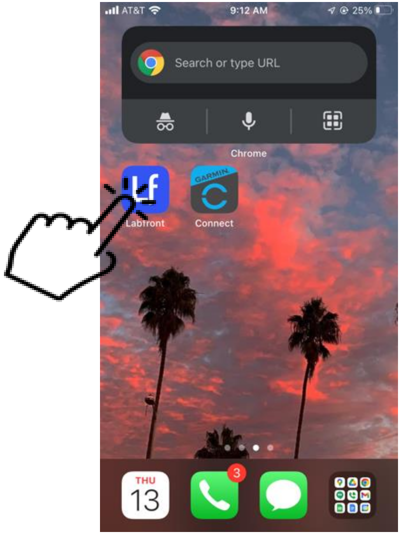

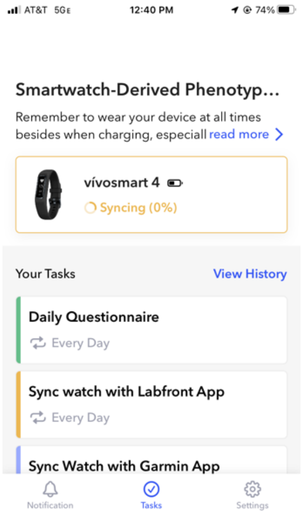

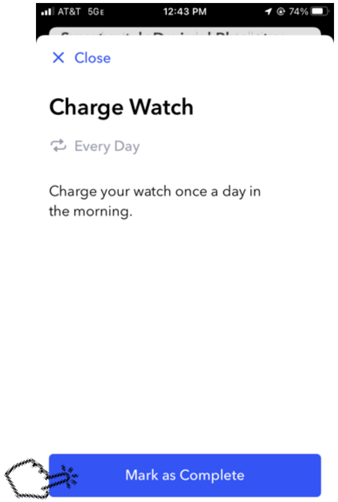

Task #2-4: Syncing with Apps and Completing Daily Survey Night

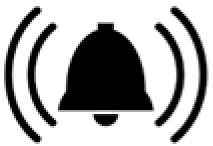

STEP 1:
Alert will appear

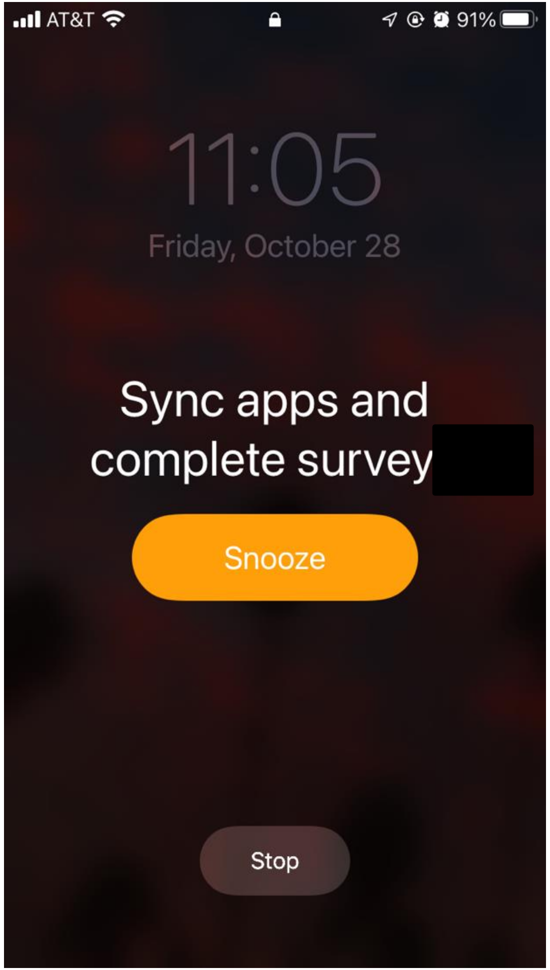

You will hear or see an alert on your phone...
Click “Stop”

If you are using your phone when you get an alert, the notification will look like this...

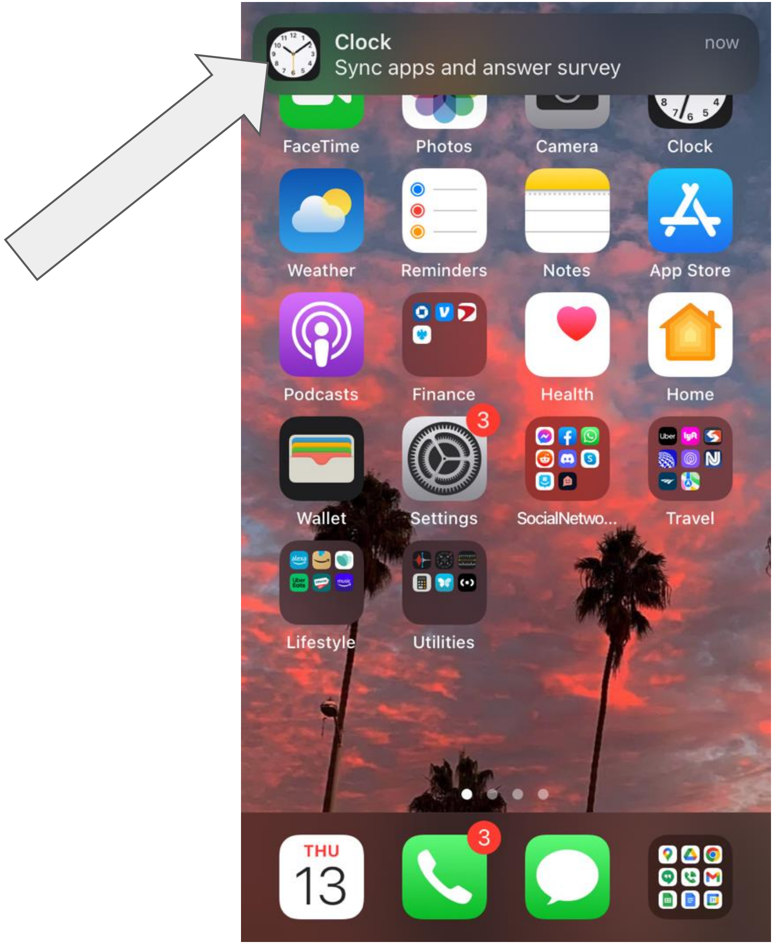

Task #2- Syncing watch with Garmin App Every Night

- Open Garmin app and then then close app, it will sync in the background. Then open Labfront to mark off that you synced with the Garmin app. Click on the "Sync with Garmin App", Then click "Mark as Complete"

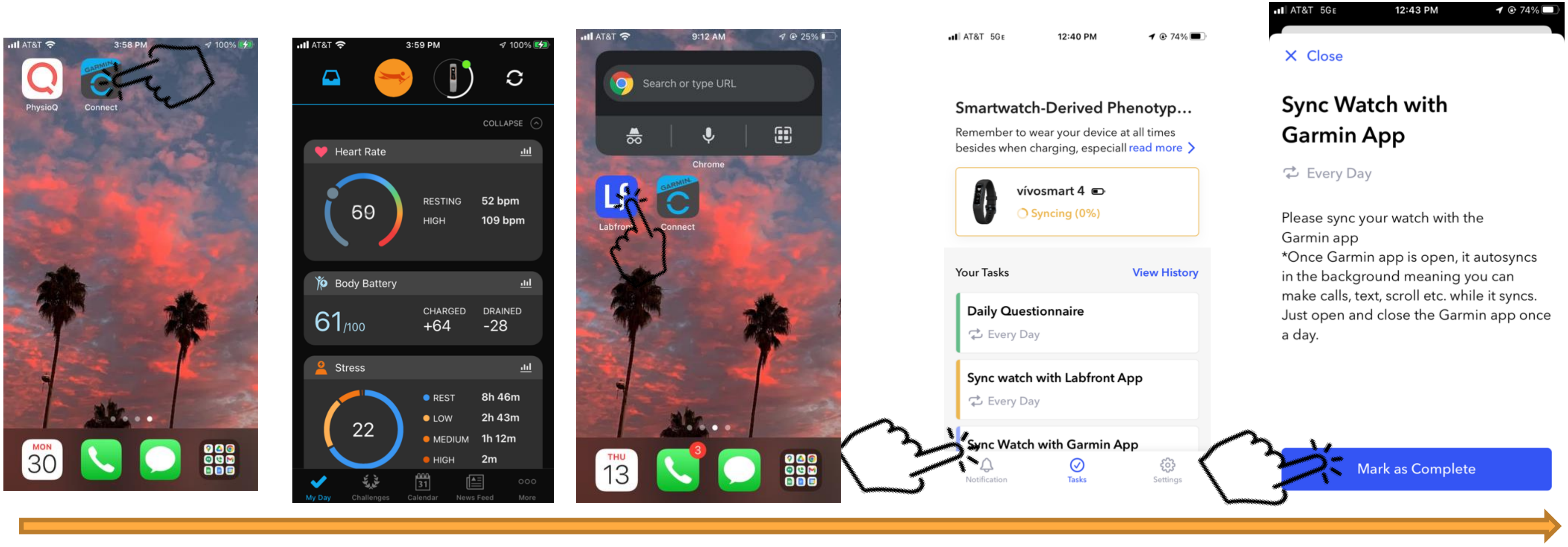

Task #3 Syncing Watch with Labfront App Every Night

- Open Labfront app and Click “Sync Now”. Then Click the task, “Sync watch with Labfront App”, then “Mark as Complete”.

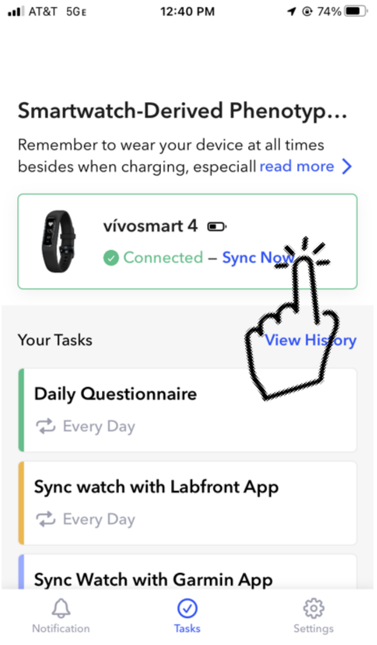

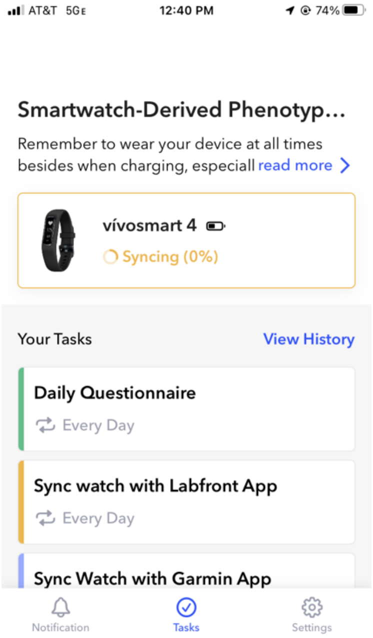

Once it says 100%, it has completed syncing. This should take under 2 minutes

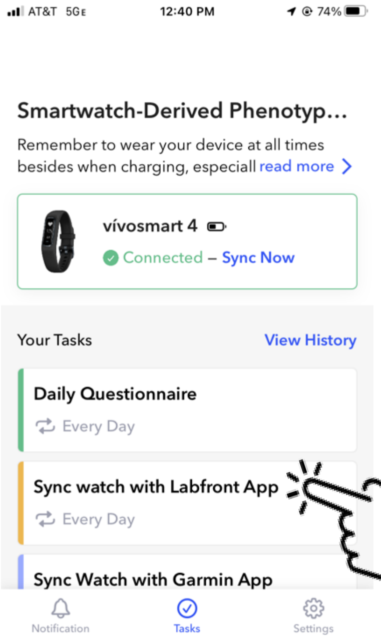

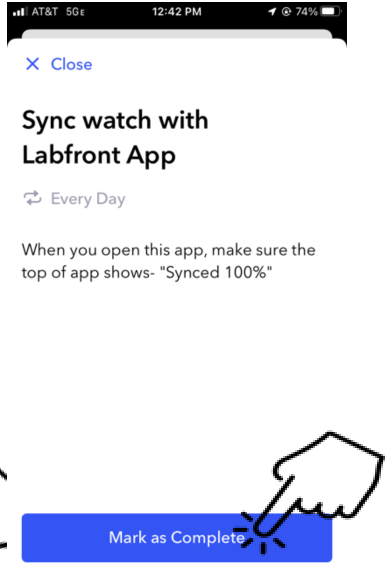

Task #4 Answering Daily Survey

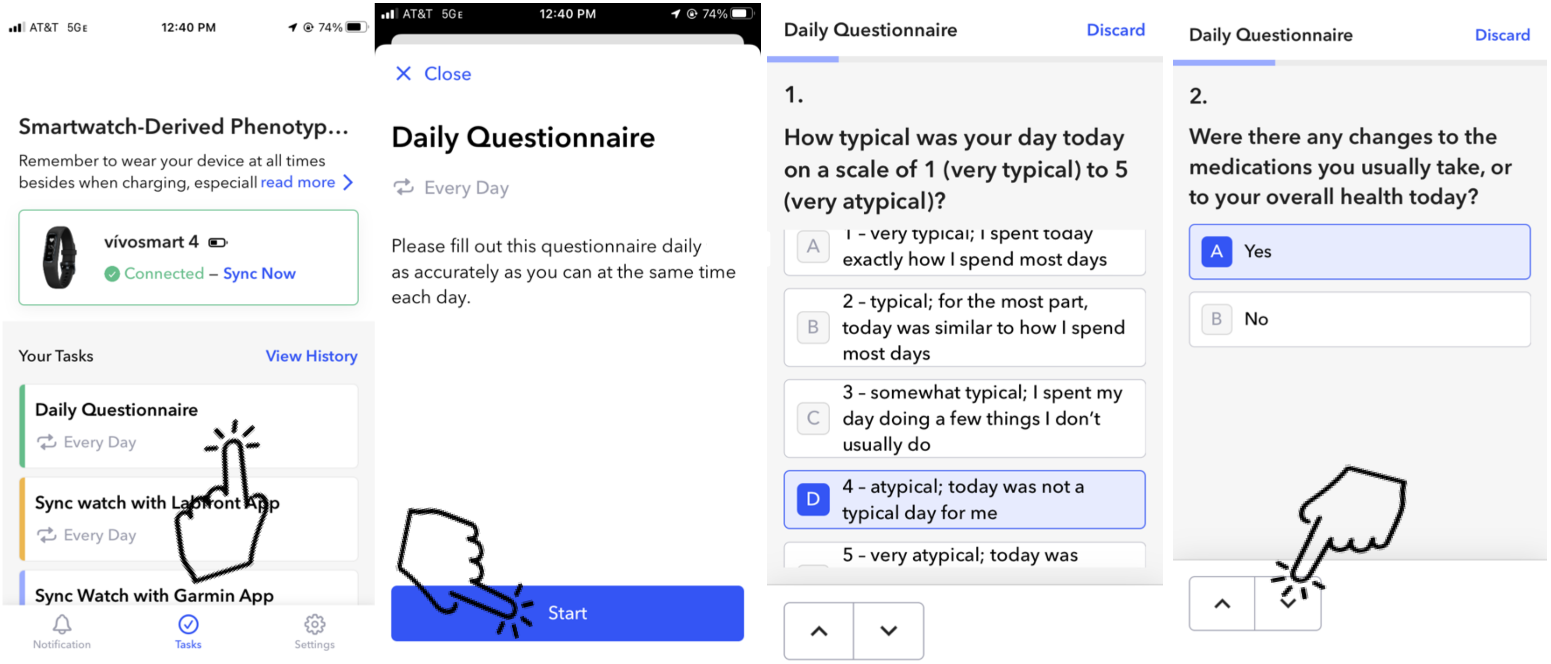

Open “Daily Questionnaire” in the Labfront App, then click “Start”

Select the answer you wish for each question then click the “Down” button

Continue Answering Survey

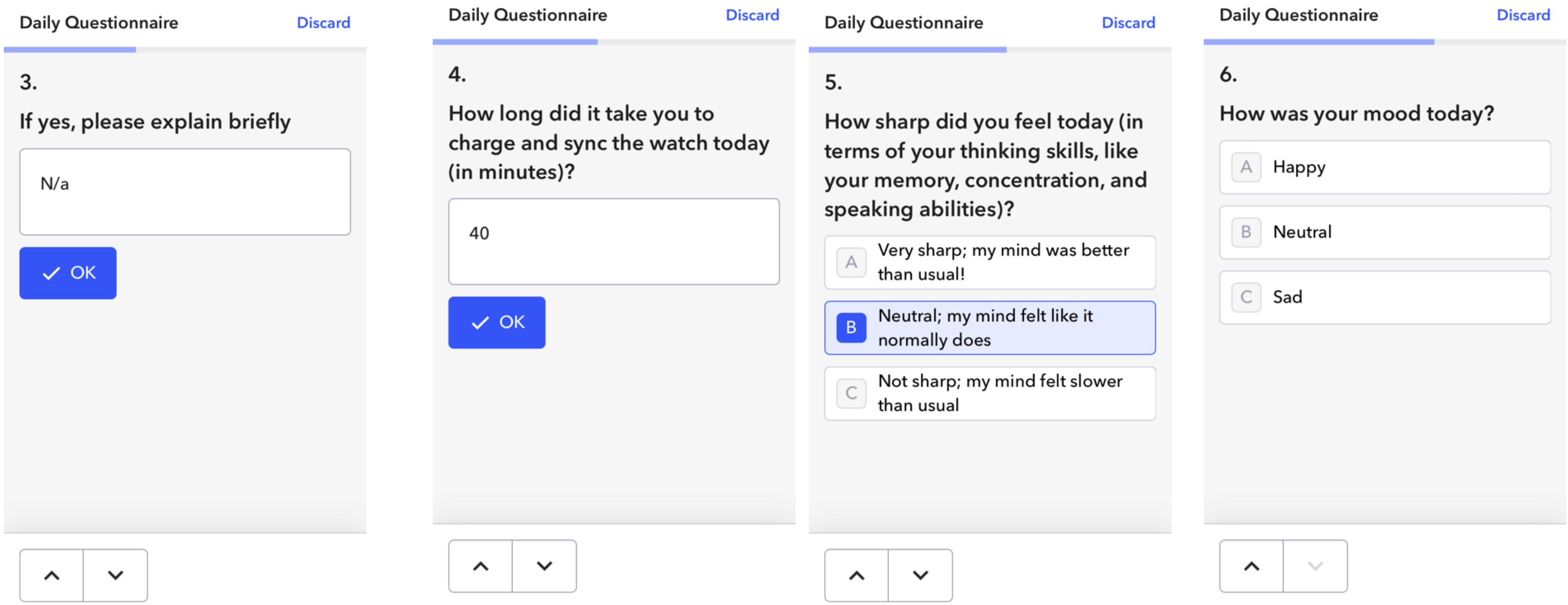

Describe changes if there were any for #3 or put N/A. If there were none then Click "OK"
Continue filling in/selecting an answer option and advancing through the survey. When you click an answer option, it will automatically advance to the next question. If you need to go back, click the up arrow at the bottom to change your answer.

Continue Answering Survey

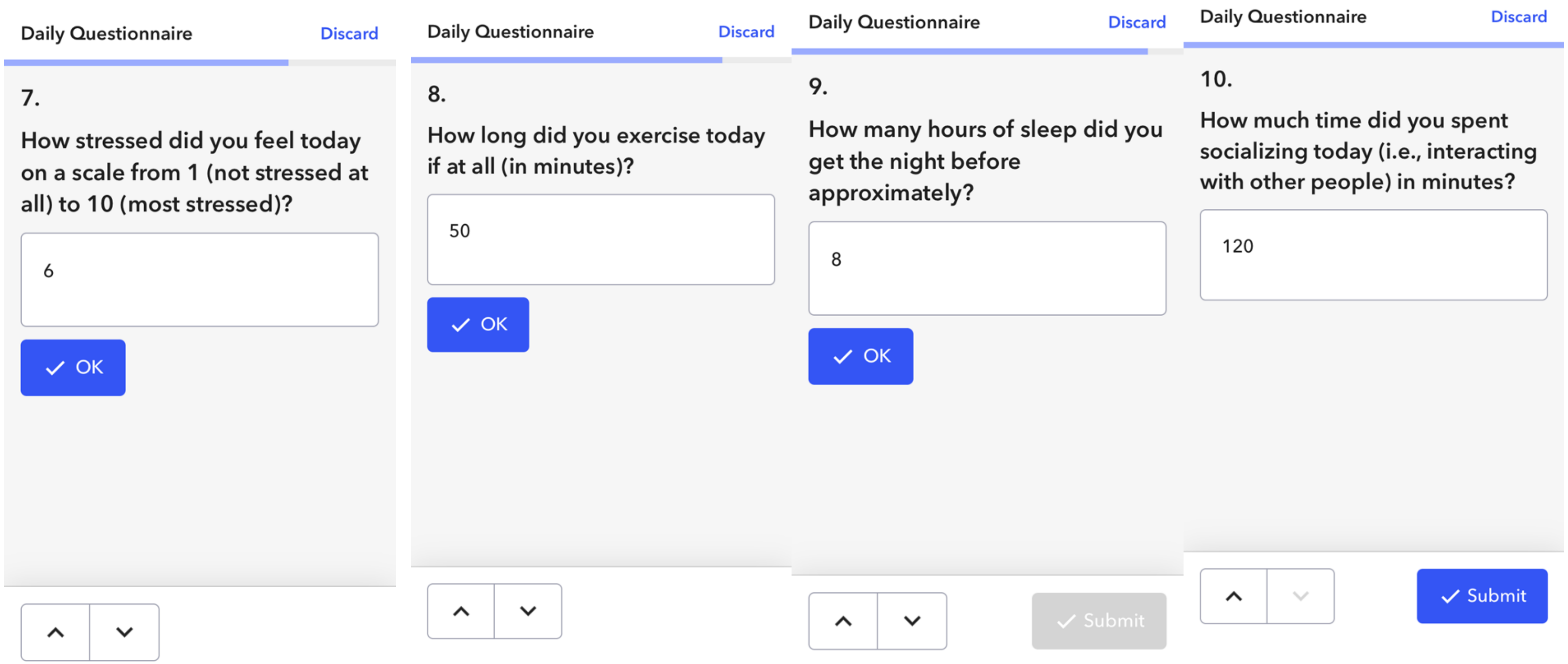

Continue filling in numbers through survey then Click “Submit” at the end.

1. Now you try it...Charging

1) Pretend it is __ am and you missed the Alarm alert
2) Show how you would charge the watch
3) Tap on the Labfront app on your phone
4) Mark the Charge watch task as complete
5) Exit app without logging out

Tell us the steps you are doing <u>out loud</u> as you do them

2. Now you try it…Syncing

1) Pretend it is __ pm and you missed the Alarm alert
2) Tap on Garmin app on your phone
3) Sync Garmin App with watch
4) Exit app without logging out
5) Tap on Labfront app on your phone
6) Mark the Garmin app Sync task in Labfront as Complete
7) While Labfront app is open, Sync Labfront App with watch
8) Mark the Labfront Sync task in Labfront as Complete
9) Exit app without logging out

Tell us the steps you are doing <u>out loud</u> as you do them

3. Now you try it... Survey

1) **Pretend it is __ pm and you missed the Alarm alert**
2) **Tap on the Labfront app on your phone**
3) **Select the Survey task**
4) **Complete each survey question**
5) **Submit the survey**
6) **Exit app without logging out**

Tell us the steps you are doing <u>out loud</u> as you do them

**Experimenter: note the number of times you repeated this step*

Just a few more points...

- What to do if you miss a survey
- Important troubleshooting reminders
- What to do if you have trouble

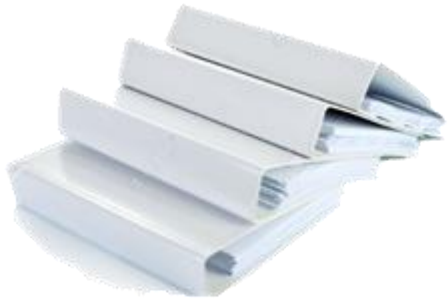

Troubleshooting Steps

- ❑ Is the watch consistently charged?
- ❑ Are you logged into Garmin and Labfront?

Look in the study binder for directions.

Still Need Help? Contact us any time.

Email: **aging@temple.edu**

Phone number: **(484)-843-1321**

Examiner: record end time
(once training is completed)

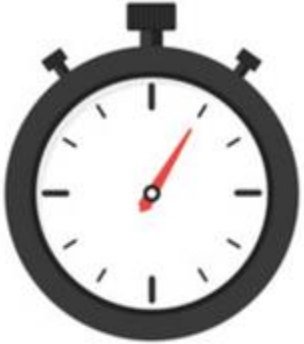

Supplement: Multimedia Appendix 1 [file humanfactors_v12i1e69952_app1.pdf]
